# Differentiating Autoimmune Hepatitis From Sulfasalazine Toxicity: The Value of Clinicopathologic Correlation

**DOI:** 10.7759/cureus.104339

**Published:** 2026-02-26

**Authors:** Adam Zoubi, Bibek Bakhati

**Affiliations:** 1 Internal Medicine, Christus Spohn Hospital, Corpus Christi, USA; 2 Internal Medicine, Texas Tech University Health Sciences Center (Permian Basin), Odessa, USA

**Keywords:** autoimmune vs drug-induced liver damage, drug-induced liver injury (dili), drug-induced transaminitis, sulfasalazine, sulfasalazine induced hepatotoxicity, sulfasalazine-induced hepatotoxicity

## Abstract

Sulfasalazine is one of the commonly used disease-modifying anti-rheumatic drugs (DMARDs) for many inflammatory and autoimmune illnesses. Sulfasalazine is generally safe with consistent monitoring, as it can rarely cause clinically significant drug‑induced liver injury (DILI). Diagnosing DILI can be challenging in patients with autoimmune diseases due to positive baseline autoantibodies that may mimic autoimmune hepatitis (AIH) or lupus‑associated hepatitis. This case highlights severe hepatocellular DILI temporally associated with sulfasalazine exposure in a patient with Sjögren's disease and systemic lupus erythematosus (SLE), where positive antinuclear antibody (ANA) could have misleadingly suggested an autoimmune liver disease. Clinical course, targeted serologies, imaging, and histology supported sulfasalazine‑induced liver toxicity rather than autoimmune or viral hepatitis. Early recognition and discontinuation of the drug were associated with rapid clinical and biochemical improvement in liver function.

## Introduction

Sulfasalazine is a sulfonamide‑containing agent widely used for ulcerative colitis and inflammatory arthritis, including rheumatoid arthritis. Hepatotoxicity from sulfasalazine is uncommon but clinically significant because it may progress to acute liver failure. Reported injury patterns include hepatocellular or cholestatic, typically within the first several weeks of therapy, and most cases resolve rapidly with discontinuation of the drug [1,2].

Drug-induced liver injury (DILI) remains a diagnosis of exclusion and can mimic other liver diseases. This diagnostic challenge is amplified in patients with systemic autoimmune disorders, such as Sjögren's disease and systemic lupus erythematosus (SLE), where baseline antinuclear antibody (ANA) positivity and immune‑mediated symptoms may raise suspicion for autoimmune hepatitis (AIH) or lupus‑associated hepatitis. Current guidance emphasizes careful exclusion of competing etiologies, assessment of temporal relationship to medication exposure, and recognition that histology may be suggestive but is often not pathognomonic [3,4].

In this case, we discuss severe, predominantly hepatocellular transaminitis temporally associated with sulfasalazine in a young female patient with Sjögren's disease, rheumatoid arthritis, and SLE, where the principal diagnostic question was distinguishing DILI from autoimmune or viral hepatitis using serologic testing, imaging, and liver biopsy.

## Case presentation

A 29‑year‑old woman with a history of Sjögren's disease, rheumatoid arthritis, and SLE presented with three days of intermittent periumbilical abdominal pain, described as stabbing and occurring on and off without clear aggravating factors. She denied nausea, vomiting, constipation, diarrhea, melena, hematochezia, yellowing of her skin/eyes, dysuria, fever, chills, or night sweats. She reported long‑term intermittent use of low‑dose prednisone as needed. She had previously been treated with disease‑modifying agents, including methotrexate and hydroxychloroquine, years ago. She had been on and off sulfasalazine, which had been discontinued in the past due to abdominal pain. However, she restarted sulfasalazine approximately three weeks prior to presentation and stopped it again on the day of presentation due to symptoms.

Physical exam showed the patient was appropriate to her documented age, not in distress, and resting comfortably. She was alert and oriented x4 and participating in providing a full history. No jaundice or scleral icterus was noted, and there was abdominal tenderness on palpation in the epigastric and right upper quadrant area, with a negative Murphy’s sign. Her bowel movement sounds were normal. Otherwise, auscultation of the respiratory and cardiac systems was normal, without lower extremity edema and without rashes or skin changes noted.

Laboratory work was done, including a comprehensive metabolic panel (CMP), as shown in Table 1, a complete blood count (CBC) in Table 2, and a coagulation profile in Table 3.

**Table 1:** Comprehensive metabolic panel A comprehensive metabolic panel was significant for elevated aspartate aminotransferase (AST), alanine aminotransferase (ALT), and alkaline phosphatase ALP with normal total bilirubin. AST: Aspartate aminotransferase, ALT: Alanine aminotransferase, ALP: Alkaline phosphatase, BUN: Blood urea nitrogen, HCG: Human chorionic gonadotropin, SGOT: Serum glutamic-oxaloacetic transaminase

Test name	Value	Ref range and unit
Glucose	78	75-105 mg/dL
BUN	9	7-19 mg/dL
Creatinine	0.6	0.60-1.10 mg/dL
Sodium	138	136-145 mmol/L
Potassium	4.2	3.5-5.1 mmol/L
Chloride	107	98-107 mmol/L
Carbon Dioxide	22	22-29 mmol/L
Anion Gap	9	6-18 mmol/L
Calculated BUN/Creat	15	mg/dL
Calcium	9.3	8.4-10.2 mg/dL
Protein Total	8	6.4-8.3 g/dL
Albumin	3.4	3.5-5.0 g/dL
SGOT (AST)	1,849 (H)	5-34 U/L
ALT	2,372 (H)	0-55 U/L
Alkaline Phosphatase	227 (H)	40-150 U/L
Bilirubin, Total	1.2	0.2-1.2 mg/dL
eGFRcr SERPlBld	124.53	>=60.00 mL/min/1.73 m^2^
A/G Ratio	0.7 (L)	>1.2 to <2.2 ratio
HCG	Negative	Negative
Acetaminophen Level	<3.0 Low	10.0-30.0 ug/mL
Thyroid-Stimulating Hormone	0.48	0.35-4.94 uIU/mL

**Table 2:** Complete blood count A complete blood count (CBC) was within the normal limit. WBC: White blood cells, RBC: Red blood cells, MCV: Mean corpuscular volume, MCH: Mean corpuscular hemoglobin, MCHC: Mean corpuscular hemoglobin concentration, RDW: Red cell distribution width, MPV: Mean platelet volume

Test name	Value	Ref range and unit
WBC	4.8	4.8-10.8 x 10^3^/uL
RBC	4.72	4.20-5.40 x 10^6^/uL
Hemoglobin	14.2	12.0-16.0 g/dL
Hematocrit	43.5	37.0-47.0%
MCV	92.2	81.0-99.0 fL
MCH	30.1	26.0-34.0 pg
MCHC	32.6	31.0-37.0 g/dL
RDW	15.7 (H)	11.2-14.0%
Platelet Count	269	130-400 x 10^3^/uL
MPV	9.5	7.4-10.4 fL
Nucleated Red Blood Cell Auto	0	0.00-0.01 x 10^3^/uL
Neutrophils %	64.8	40.0-78.0%
Lymphocytes %	16.9	15.0-48.0%
Monocytes %	15.4 (H)	0.0-12.0%
Eosinophils %	1.5	0.0-7.0%
Basophils %	0.6	0.0-3.0%
Neutrophils Absolute	3.11	1.90-8.00 x 10^3^/uL

**Table 3:** Coagulation testing The coagulation test results were within the normal limits. INR: International normalized ratio, PTT: Partial thromboplastin time

Test name	Value	Ref range and unit
INR	1	<=4.5 ratio
Prothrombin time	11.4	9.4-12.5 s
PTT	33.6	25.0-37.0 s

Due to the CMP revealing significant transaminitis (Table 1) and a history of autoimmune diseases, as mentioned above, a hepatitis panel (Table 4) and an autoimmune panel (Table 5) were ordered.

**Table 4:** Acute hepatitis panel The result of the acute hepatitis panel was negative. HCV: Hepatitis C virus, CMV: Cytomegalovirus, HDV: Hepatitis D virus, NAAT: Nucleic acid amplification test, PCR: Polymerase chain reaction

Test name	Value	Ref range and unit
Hepatitis A IgM Antibody	Non-reactive	Non-reactive
Hepatitis B Surface Antigen	Non-reactive	Non-reactive
Hepatitis B Core IgM Antibody	Non-reactive	Non-reactive
Hepatitis C Ab	Non-reactive	Non-reactive
HCV Qnt by NAAT (IU/mL)	Not Detected	Not Detected
HCV Qnt by NAAT (log IU/mL)	Not Detected	Not Detected
HCV Qnt by NAAT Interp	Not Detected	Not Detected
CMV IgM Antibody	<8.0	<=29.9 AU/mL
HDV by Quantitative PCR, Interp	Not Detected	Not Detected
HDV by Quantitative PCR, IU/mL	<92	IU/mL
HDV by Quantitative PCR, Log IU/mL	<2.0	log IU/mL

**Table 5:** Autoimmune panel The autoimmune panel result was consistent with the known medical history of SLE and Sjogren's disease, as well as with autoimmune studies done in the past. ELISA: Enzyme-linked immunosorbent assay, IgG: Immunoglobulin G, RNP Ab: Ribonucleoprotein antibody, Anti-JO-1: Anti-histidyl tRNA synthetase, SS-B/La Ab: Anti-Sjögren's syndrome B/Anti-La, ANA: Antinuclear antibody, ENA: Extractable nuclear antigen, SM Ab: Smooth muscle antibodies, C3: Complement 3, C4: Complement 4

Test name	Value	Ref range and unit
RNP Ab, IgG, S	12	0-19 Units
Anti-JO-1	1	0-40 AU/mL
Scleroderma SCL-70	2	0-40 AU/mL
Anti-Mitochon Ab IFA	6.4	0.0-24.9 Units
Ss-B/La Ab Igg	43 (H)	0-40 AU/mL
Anti-Nuclear Ab (ANA), IgG by ELISA	Detected	None Detected
ANA Pattern	Speckled	
ANA Titer	1:640	<1:80
Antinuclear Antibody (ANA), HEp-2, IgG	Detected (H)	None Detected
Double-Stranded DNA (dsDNA) Ab IgG ELISA	186 (H)	0-24 IU
Double-Stranded DNA (dsDNA) Ab IgG IFA	1:320 (H)	<1:10
Smooth Muscle Ab, IgG Titer	<1:20	<1:20
SSA-52 (Ro52) (ENA) Antibody, IgG	90 (H)	0-40 AU/mL
SSA-60 (Ro60) (ENA) Antibody, IgG	81 (H)	0-40 AU/mL
SM Antibody	4	0-40 AU/mL
IgG 1	752	240-1118 mg/dL
IgG 2	368	124-549 mg/dL
IgG 3	117	21-134 mg/dL
IgG 4	20	1-123 mg/dL
C3 Complement	145	83-193 mg/dL
C4 Complement	28	15-57 mg/dL
Histone Ab, IgG	4.2 (H)	0.0-0.9 Units
Liver Cytosolic Type 1 IgG	Negative	Negative
Liver-Kidney Microsome-1 Ab, IgG by ELISA	2.3	0.0-24.9 U
Ribosomal P Antibody	<0.2	<1.0 AI

**Figure 1:**
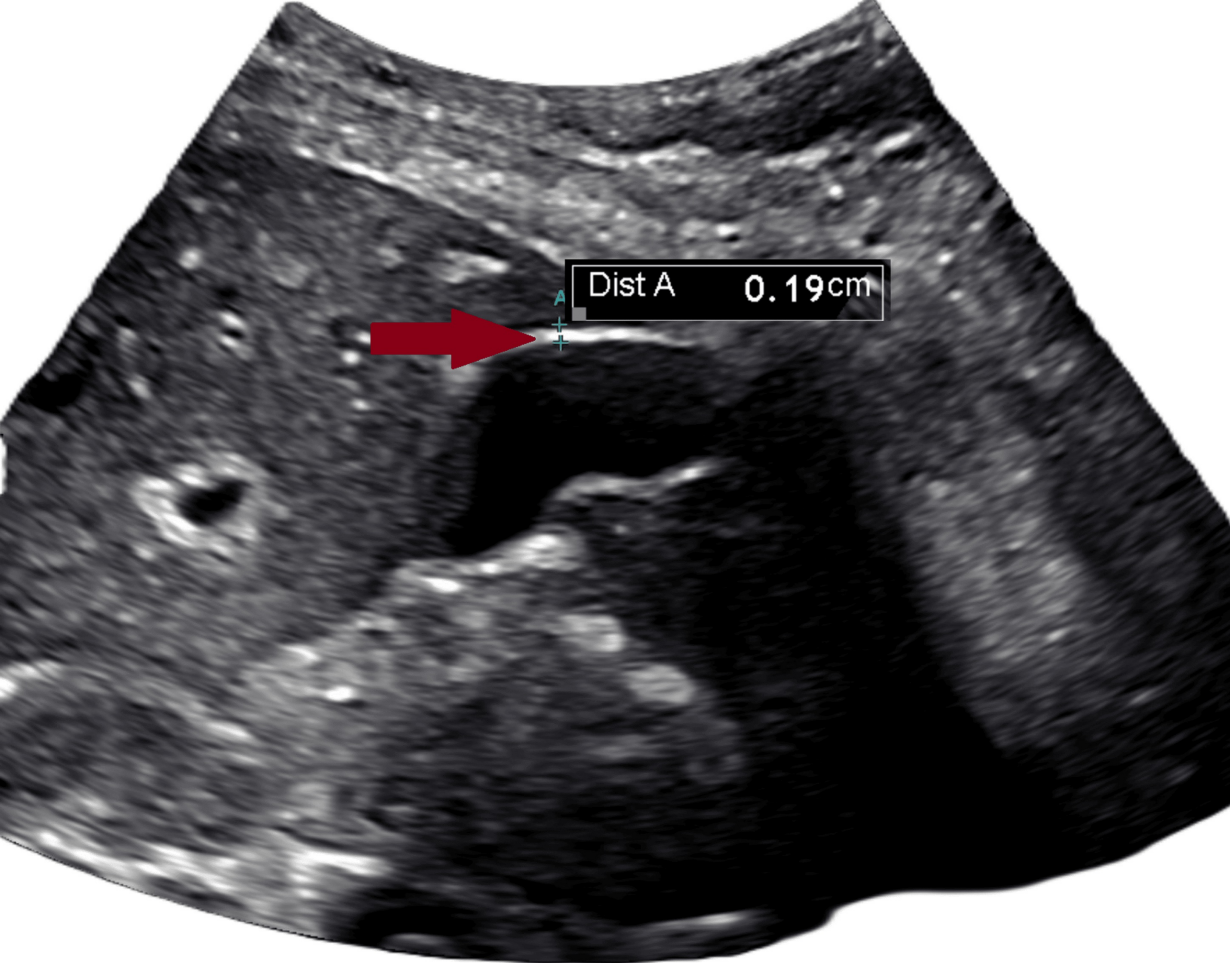
Right upper quadrant ultrasound image of the gallbladder The right upper quadrant ultrasound image (RUQ US) shows a normal gallbladder wall (red arrow), measuring 1.9 mm without thickening.

**Figure 2:**
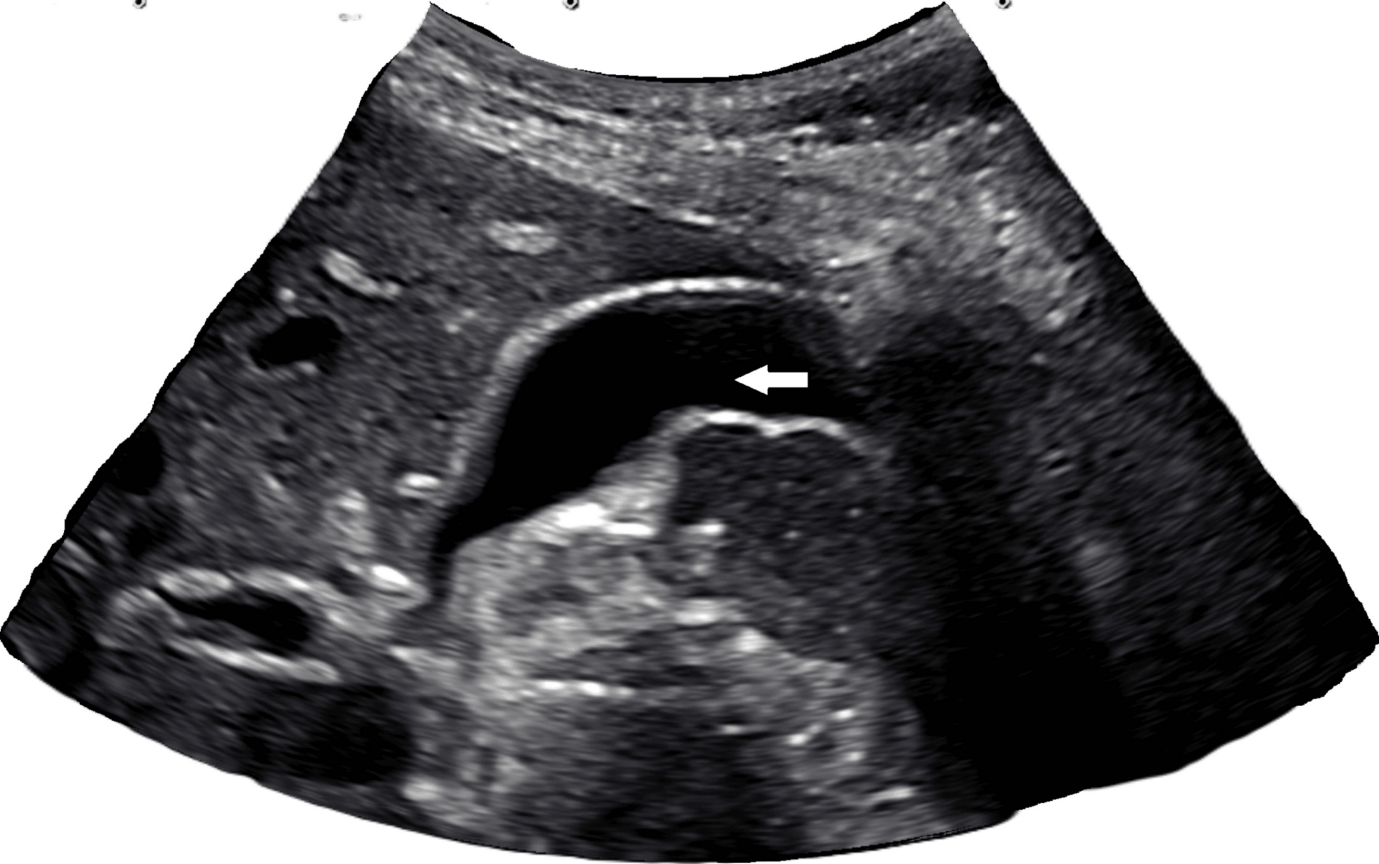
Right upper quadrant abdomen ultrasound of the gallbladder The right upper quadrant abdomen ultrasound of the gallbladder shows a normal gallbladder (arrow) without stones or dilatation.

**Figure 3:**
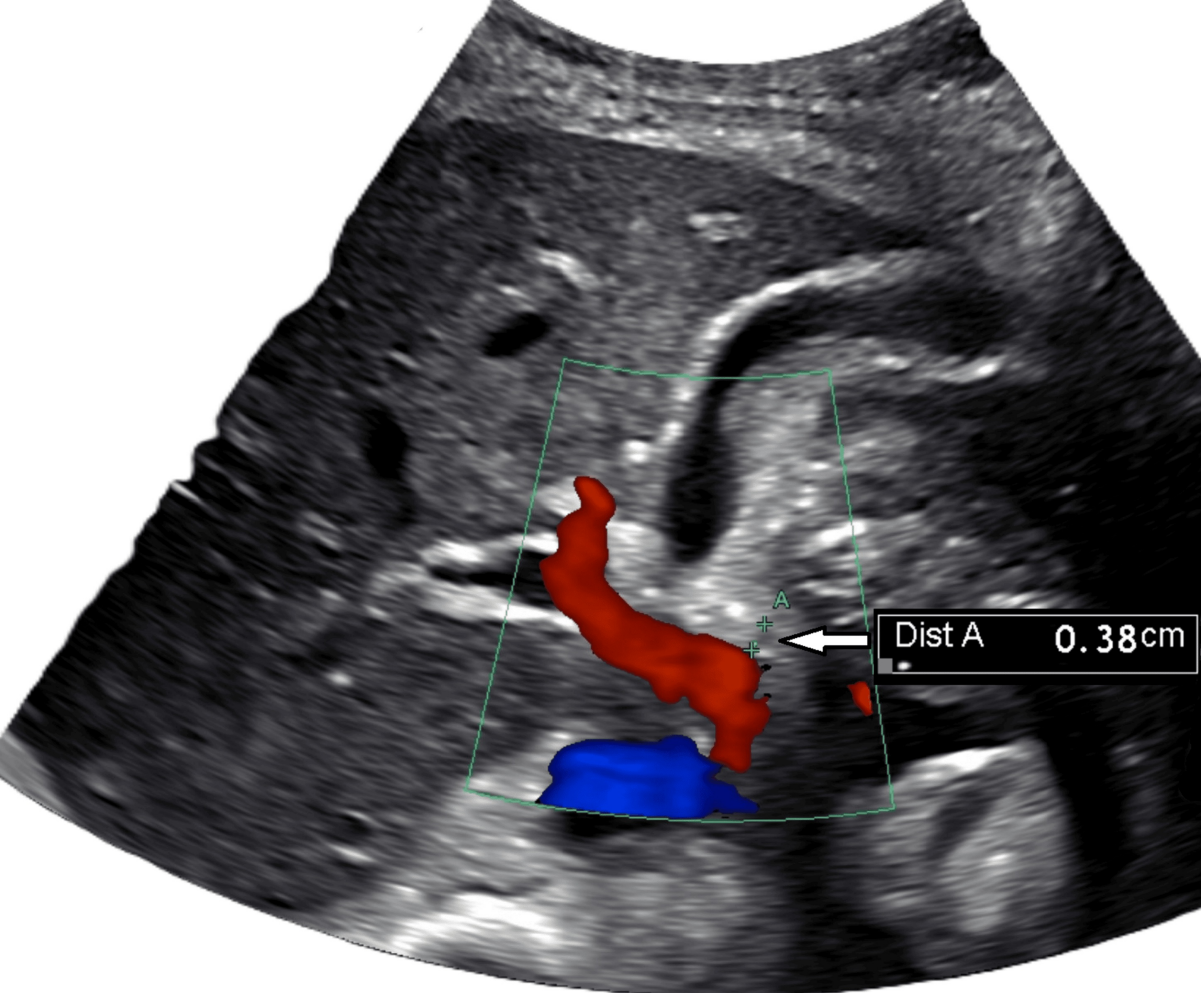
Right upper quadrant abdomen ultrasound of the common bile duct The right upper quadrant abdomen ultrasound shows a common bile duct (arrow) measuring 3.8 mm without dilatation or stones.

Figures 1-3 show the abdominal ultrasound of the right upper quadrant, which was negative for any structural changes.

**Figure 4:**
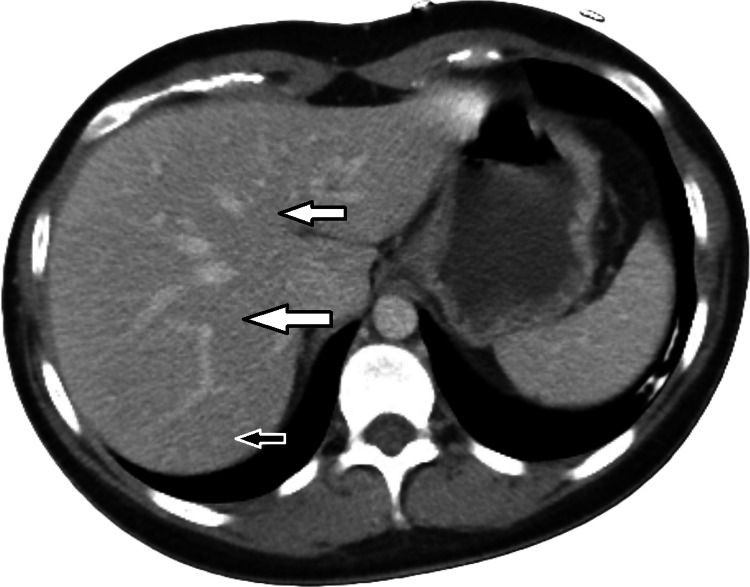
Computed tomography (CT) abdomen and pelvis with IV contrast Computed tomography (CT) abdomen and pelvis with intravenous contrast shows normal hepatic biliary ducts without dilatation (arrows).

**Figure 5:**
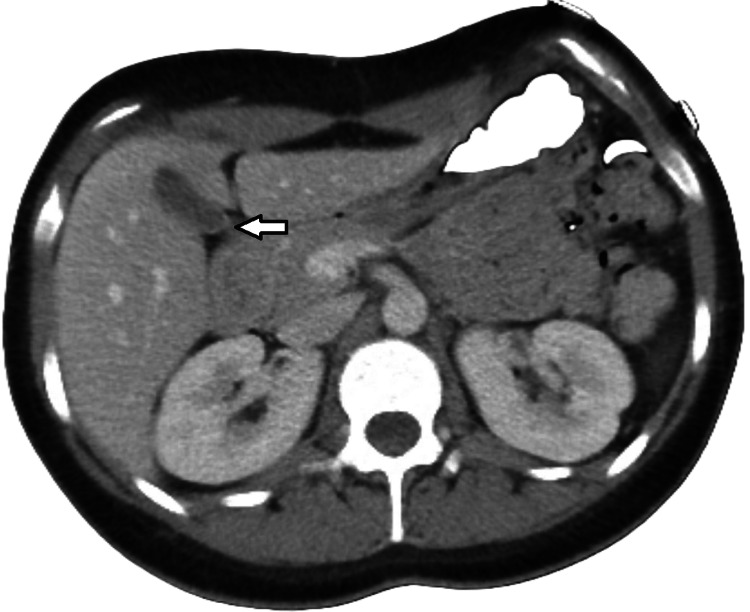
Computed tomography (CT) abdomen and pelvis with intravenous contrast Computed tomography (CT) abdomen and pelvis with intravenous contrast shows a normal gallbladder without stones or dilatation.

An abdomen and pelvis CT scan with IV contrast was also negative for any structural changes, as shown in Figures 4-5.

**Figure 6:**
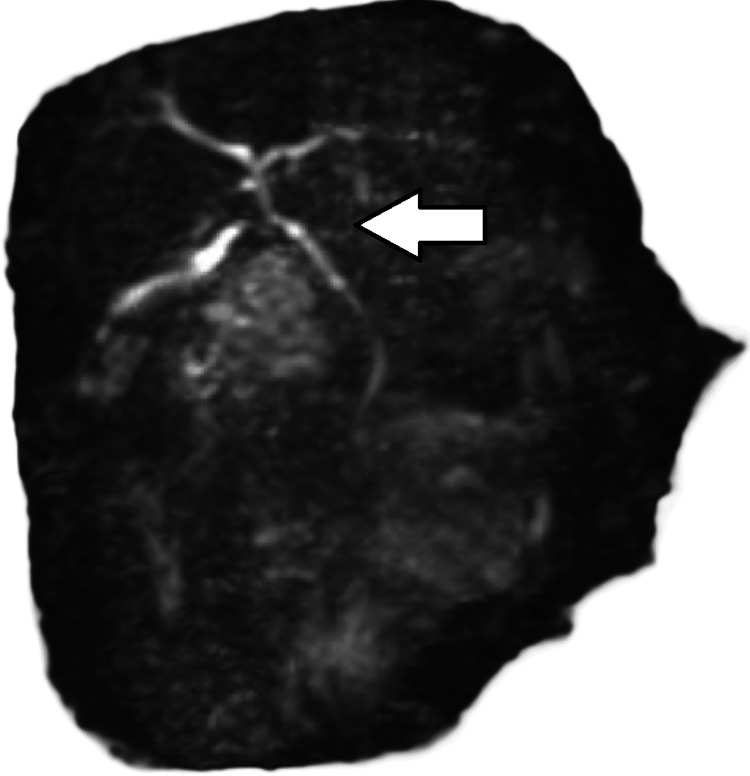
Magnetic resonance cholangiopancreatography (MRCP) abdomen without contrast MRCP with maximum intensity projection (MIP) shows that the common bile duct is not dilated and no filling defects are seen. The common bile duct measures up to 2 mm in diameter (arrow).

**Figure 7:**
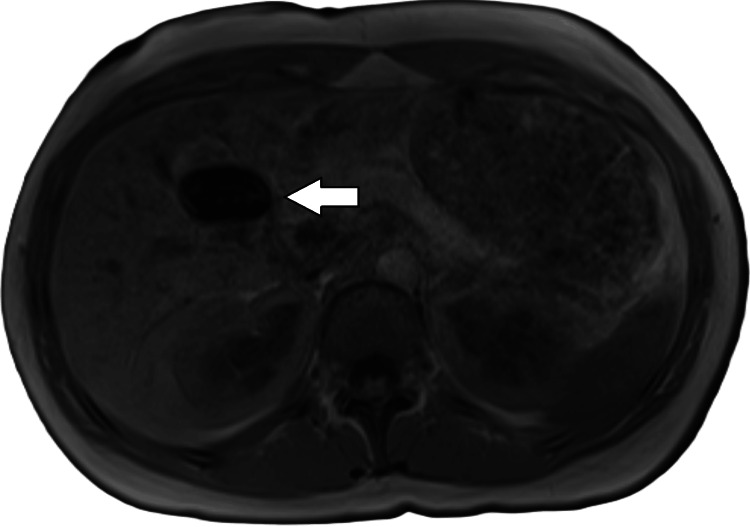
MRI abdomen without contrast MRI abdomen without contrast (MRCP) shows that the gallbladder is present and not distended (arrow).

MRI abdomen without contrast (magnetic resonance cholangiopancreatography, MRCP) was negative, as shown in Figures 6-7.

A gastroenterologist was consulted and recommended holding off on starting any steroids until diagnosis is established and hydration and serial liver function tests are done. N-acetylcysteine (NAC) was not given due to normal PT/INR and no encephalopathy indicating acute liver failure. A liver biopsy was also scheduled and done during admission.

**Figure 8:**
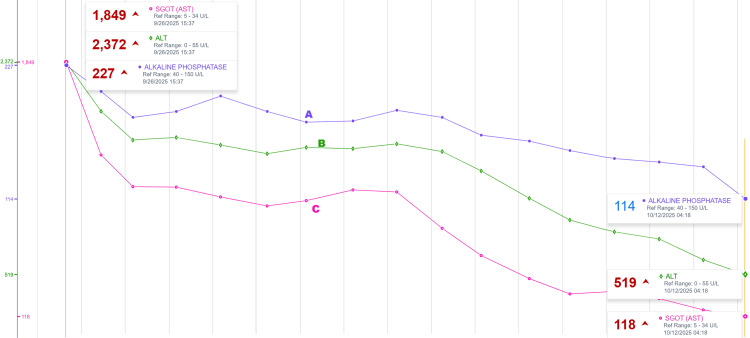
Trend of the liver function test throughout admission Trend of the liver function test throughout admission shows significant improvement while being monitored daily in-patient. Alkaline phosphatase (ALP) trend demonstrated with a purple line and letter A: showing that ALP went down from 227 U/L on admission day, down to 114 U/L on the day of discharge. Alanine transaminase (ALT) trend demonstrated with a green line and letter B: showing that ALT went down significantly from 2,372 U/L on admission day, down to 519 U/L prior to discharge. Aspartate aminotransferase (AST) trend demonstrated with a ping line and letter C: showing that AST trended down from 1,849 U/L on admission day, down to 118 U/L on discharge day. The initial significant and rapid improvement is likely due to supportive management with IV fluids and stopping sulfasalazine. SGOT: Serum glutamic-oxaloacetic transaminase

Throughout admission, daily liver function tests showed gradual improvement of aspartate aminotransferase (AST), alanine transaminase (ALT), and alkaline phosphatase (ALP), as shown in Figure 8.

**Figure 9:**
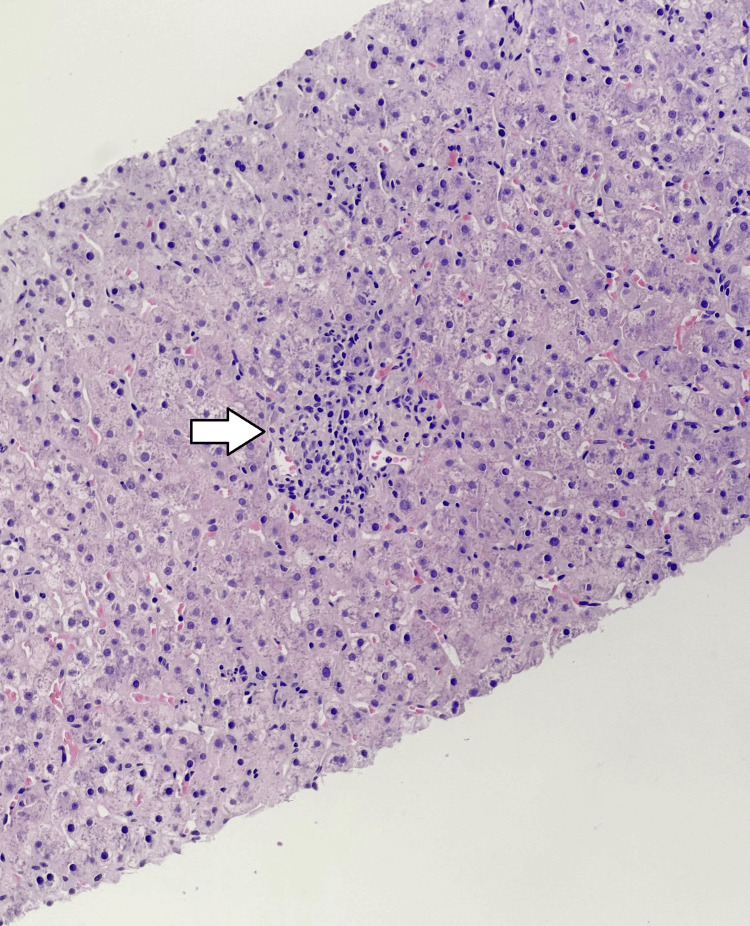
Histopathology of the liver biopsy Minimal-to-mild lobular inflammation composed mainly of lymphocytes (arrow).

**Figure 10:**
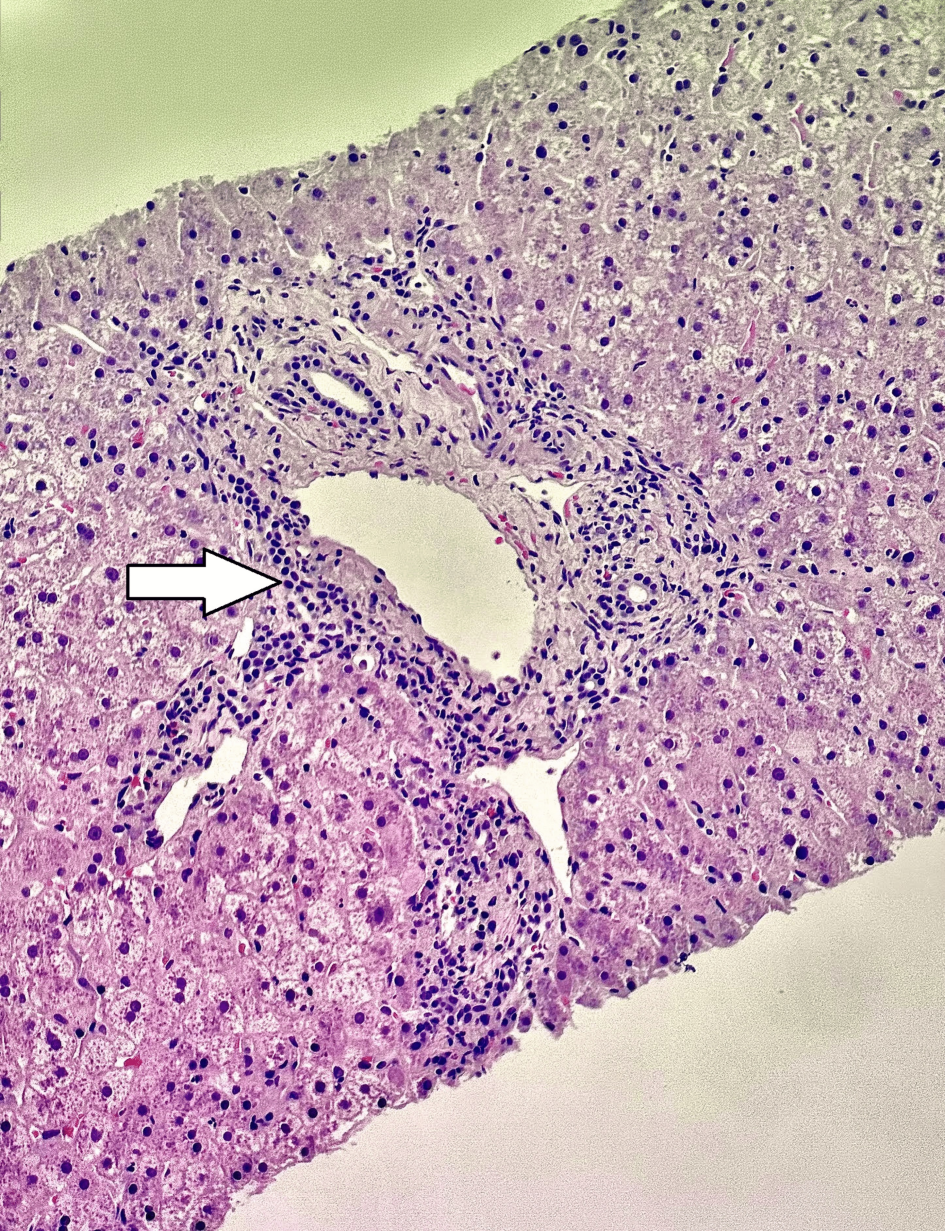
Histopathology of the liver biopsy Minimal-to-mild portal inflammation (arrow).

The liver biopsy (Figures 9-10) was adequate for interpretation (stain quality adequate; >10 portal tracts). It showed a resolving hepatitis pattern with minimal-to-mild portal and lobular inflammation composed mainly of lymphocytes, with occasional plasma cells, rare eosinophils, and numerous ceroid-laden macrophages, along with frequent acidophil bodies. There was no significant interface activity, no bridging necrosis or parenchymal collapse, and only mild bile ductular proliferation; the bile ducts, hepatic arterioles, and portal vein branches were intact, with no bile duct lesions or granulomas. Special stains demonstrated no abnormal fibrosis on trichrome, no hemosiderosis on iron stain, and a relatively intact reticulin framework without features of nodular regenerative hyperplasia. Periodic acid-Schiff with diastase (PAS-D) was negative for periportal alpha-1 antitrypsin globules and highlighted the ceroid-laden macrophages; CK7 highlighted bile ducts/ductules without aberrant periportal hepatocyte expression; and the copper stain was negative. Viral studies were negative, including cytomegalovirus (CMV) immunohistochemistry, herpes simplex virus (HSV)-1/2 immunohistochemistry, and Epstein-Barr encoding region (EBER) in situ hybridization (EBER ISH). Overall, the findings were interpreted as nonspecific mild portal and lobular inflammation consistent with resolving hepatitis, most commonly seen with a drug/toxin reaction (including herbal/dietary supplements) or, less likely, a viral infection, and there were no histologic features of overt AIH, chronic biliary tract disease, steatohepatitis, or venous outflow impairment.

Clinically, the patient's abdominal pain improved as well, she remained clinically stable without systemic symptoms, and she was discharged with immediate outpatient follow‑up with her primary care physicians (PCPs) and rheumatologist to establish a new safe treatment regimen for her extensive autoimmune diseases and continued follow-up of biochemistry and liver function profile.

## Discussion

This case illustrates a diagnostic challenge: acute transaminitis in a young woman with multiple autoimmune diseases shortly after restarting a medication that is uncommonly leading to hepatotoxicity.

The major competing considerations were (1) sulfasalazine‑induced DILI, (2) AIH or lupus‑associated hepatitis, and (3) viral hepatitis or other inflammatory/infectious causes.

Sulfasalazine-induced DILI was the highest on differential due to the following: (1) Clear timeline of the drug initiation and clinical presentation: The patient restarted sulfasalazine and developed symptoms and marked hepatocellular injury within ~3 weeks [1], fitting the recognized window in which sulfasalazine can trigger hepatic injury. Sulfasalazine hepatotoxicity commonly occurs within the first several weeks of treatment and may be severe, leading to acute liver failure. (2) Liver function biochemical improvement after discontinuation: Most sulfasalazine hepatotoxicity cases resolve after medication withdrawal [2], often within weeks (longer if cholestasis is prominent). This patient's AST/ALT declined substantially over the following days after cessation, and only supportive management without steroids or adding other medications was provided [3]. (3) Predominantly hepatocellular injury pattern with minimal bilirubin elevation: The initial ALT/AST were strikingly high, while bilirubin remained only mildly elevated, and imaging showed no obstruction or other structural changes that could explain the etiology for this presentation. This is compatible with acute hepatocellular DILI [1,2]. (4) Lastly and most importantly, histology findings were interpreted as nonspecific mild portal and lobular inflammation consistent with resolving hepatitis, most commonly seen with a drug/toxin reaction (including herbal/dietary supplements) [3-5] or, less likely, a viral infection. However, viral hepatitis was less likely due to negative viral hepatitis panels, and there were no histologic features of overt AIH, chronic biliary tract disease, steatohepatitis, or venous outflow impairment.

Autoimmune hepatitis or lupus hepatitis was less likely. This patient's baseline autoimmune conditions complicated the interpretation because ANA positivity can occur independent of liver disease. However, multiple features were inconsistent with AIH/lupus hepatitis as the primary cause for our patient presentation: (1) Autoantibodies more specific for AIH were negative (i.e., anti‑smooth muscle antibody, liver-kidney microsomal type 1 (LKM-1), and liver cytosol type 1 (LC-1) [5]. The ribosomal P antibody, which is often associated with lupus hepatitis [6], was negative. (2) A liver biopsy did not show AIH changes: Absence of any significant interface hepatitis and a plasma cell‑rich infiltrate and absence of any fibrotic changes [5,6]. (3) Improvement without steroids and only after stopping sulfasalazine favors DILI.

Viral hepatitis and structural disease were less likely. (1) A viral hepatitis panel was negative, and a liver biopsy was not consistent with viral hepatitis changes (viral hepatitis liver biopsy often shows inflammatory cell infiltration and hepatocellular damage, cholestasis, Kupffer-cell activation, endotheliitis, bile-duct damage, the ductular reaction, and hepatocellular regeneration) [7]. (2) Imaging (RUQ US, CT, MRCP) was negative for any structural changes, ruling out obstructive or structural biliary disease. (3) No evidence of venous outflow obstruction was found on evaluation, and a biopsy did not support venous outflow impairment.

**Figure 11:**
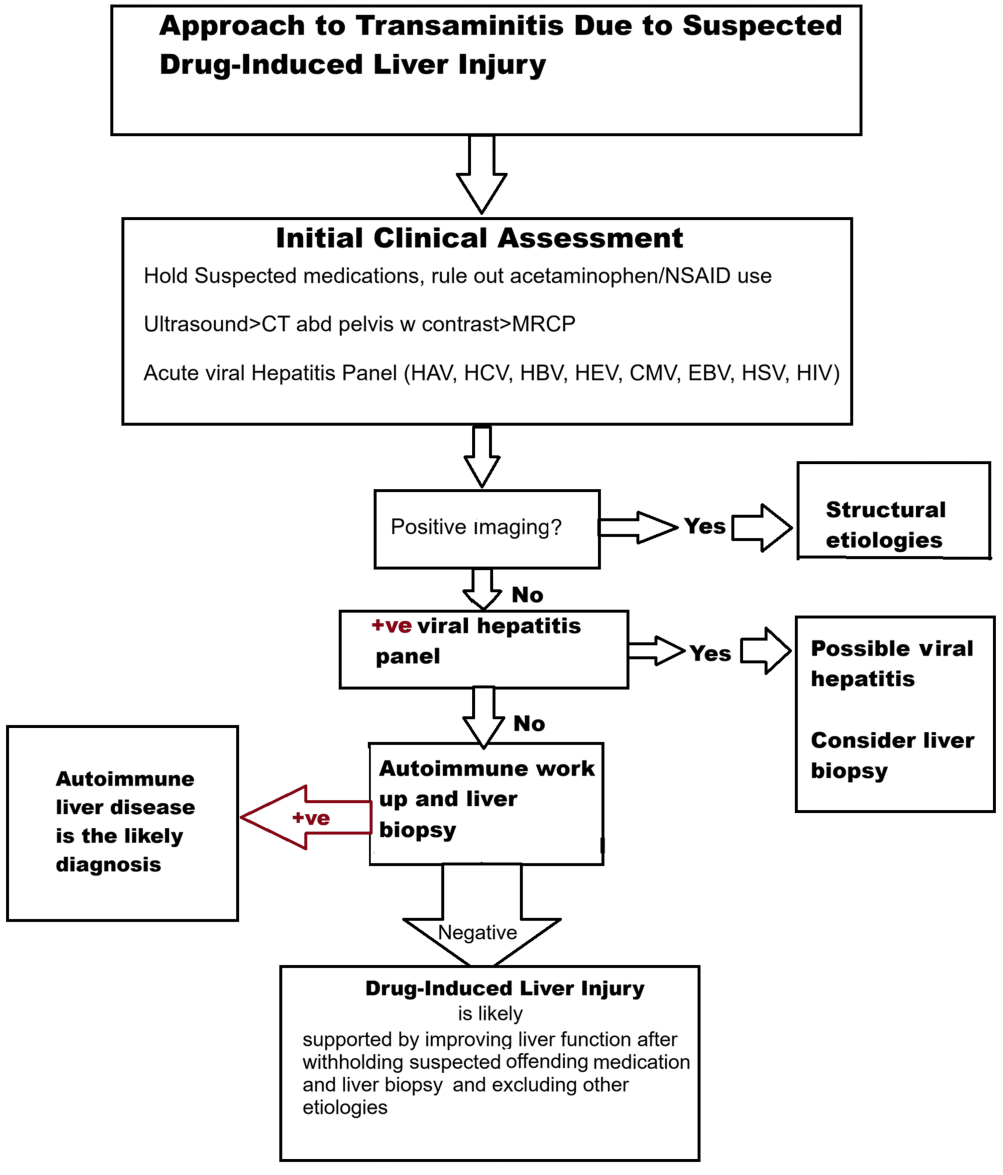
Approach to transaminitis due to suspected drug-induced liver injury CT: Computed tomography, MRCP: Magnetic resonance cholangiopancreatography, HAV: Hepatitis A virus, HCV: Hepatitis C virus, HBV: Hepatitis B virus, HEV: Hepatitis E virus, CMV: Cytomegalovirus, EBV: Epstein-Barr virus, HSV: Herpes simplex virus, HIV: Human immunodeficiency virus, +ve: positive, -ve: negative

Sulfasalazine hepatotoxicity is uncommon [8] but, potentially, can be severe and lead to acute liver failure if unrecognized with continuous exposure to the offending agent. Consider the following algorithmic approach presented in Figure 11 in such cases.

## Conclusions

In this case, we discussed a young woman with a past medical history of Sjögren's disease, rheumatoid arthritis, and SLE. She developed symptomatic hepatocellular liver injury three weeks after restarting sulfasalazine, with rapid symptomatic and liver function improvement after discontinuation of sulfasalazine and supportive management with IV fluids. Despite strongly positive autoimmune serologies, which are expected in the context of her autoimmune disease history, the absence of AIH‑associated antibodies (including smooth muscle antibody) and negative viral hepatitis workup, and her liver biopsy demonstrating resolving hepatitis without interface activity or other features of overt autoimmune hepatitis, supported a diagnosis of sulfasalazine‑induced DILI rather than autoimmune or viral hepatitis.

This case stresses the value of (1) careful medication timeline assessment, (2) targeted autoimmune/viral testing beyond ANA alone, and (3) integrating liver biopsy interpretation with clinical course to distinguish DILI from autoimmune liver disease in patients with systemic autoimmunity. This case also underscores the importance of routine and regular liver function testing when initiating sulfasalazine or similar medications for long-term use.
